# Québec Labour Confronts the Climate Crisis

**DOI:** 10.1177/0160449X251356491

**Published:** 2025-07-07

**Authors:** Ian MacDonald

**Affiliations:** 1 School of Industrial Relations, University of Montreal, Montreal, Canada

**Keywords:** climate crisis, union strategy, internal union practices, Québec, labor environmentalism

## Abstract

Most academic literature on unions and climate action is concerned with the analysis of union positions on climate as expressed by union representatives and in official documents. We as yet know little of the internal processes that lead unions to take these positions, and in particular the tensions or contradictions among labour actors that prevent unions from being more effective climate actors. This paper contributes to this body of work by exploring three areas of tension and contradiction that confront climate action within the Québec labour movement. These include tensions within unions with respect to the importance that climate action ought to occupy in union strategy, sharp disagreements on strategic and tactical questions having to do with rank-and-file organizing, industrial legality, and political demands, and an emerging contradiction between private and public sector positions on climate action and sustainable futures.

If we take seriously Matt Huber's contention (2022) that climate change is an ecological form of the class war waged by capitalists to the extreme detriment of working people on the planet, we must also take seriously as climate actors the organisations – unions – that workers have built to defend themselves in this class war. This is even more the case if we recognise that unions are located at the centre of the social process – capitalist production – that is driving the crisis. By ‘take seriously’ I mean considering unions as internally complex social organizations with capacities as well as limits that are given in the fact that they were built by workers to regulate capital in particular workplaces, and that these organizations are in turn regulated by the capitalist state in contradictory ways. There is a tendency in the rapidly growing literature on unions as climate actors to bracket this simple observation, to discuss unions as unitary actors without attending to the internal tensions and contradictions that must be worked through in the process of unions addressing the climate crisis. We should be more concerned with how the climate crisis and climate action play out within union organizations that lead to unions taking climate action. And this is important if we are to better understand the barriers to more effective climate action that are within the capacity of trade unionists to change.

In this paper I am guided by two injunctions. First, Richard Hyman cautioned in his much-cited paper on union strategic action that “action clearly occurs *within* organisations, and by individuals and groups *in the name* of organisations… but these social and political processes are mystified by attributing the essentially human process of action … to more abstract and impersonal entities” (Hyman 2007, 200). And second, Paul Hampton's plea, on observing that most research on unions and climate is based on official statements and documents, that “such a ‘top-down view will need to be supplemented with bottom-up approaches that capture the tensions and contradictions between and within … trade unions” (Hampton 2015, 191).

It remains the case, as Torsten Geelan (2024) has shown in an exhaustive survey of the literature, that most research on unions and climate change takes national and local unions as the unit of analysis, and that research methods rely primarily on documentary analysis and secondarily on interviews with union representatives. A small literature has emerged on internal union activism and dynamics around climate action, largely confined to countries sharing a social democratic tradition. Kori Allan and Joanna Robinson (2022) have recently taken up Hampton's injunction to reveal limited membership support, divided by age and employment status, for the eco-modernist positions adopted by union leadership in the Canadian auto industry : older workers with stronger employment protection are less likely to prioritize climate over other concerns, while those younger and more precariously-employed are more open to a climate jobs transition beyond the auto industry and capitalism. Ragnar Lündstrom's (2017) research in Sweden, based on biographical studies of labour climate activists at different levels of union organization, brings out the organisational constraints on climate activism within a highly institutionalised labour regime. Union structures are aligned with business and the state, and do not facilitate cross-union organising on ecological working class concerns. Johan Gärdebo's work identifies tensions between ‘emplaced’ discussions of climate at worksites and the positions adopted by labour centrals, which are “more actively engaged in initiating and implementing industrial decarbonization strategies, but had developed this priority in reaction to discourse from other policy-professionals rather than from different segments of their trade union” (Gärdebo 2023, 2). Michaud and Laroche (2024) identify this institutional cloistering of climate knowledge and activism within labour centrals as a barrier to more effective labour climate action in their study of collectively-bargained ‘green clauses’ in Québec. David Jordhus-Lier et al.'s (2024) sober assessment of the Norwegian labour movement's failure to play a decisive role in that country's decarbonisation efforts also refers to such ‘scalar’ tensions within the labour movement, set aside competing visions of just transition among unions, rooted ultimately in how they relate to fossil capital.

Here I offer a national case study of tensions and contradictions that occur at the organizational level, within unions and across the private sector/public sector divide, as unions and labour centrals in Québec confront the climate crisis. While tensions and contradictions within the house of labour are ultimately rooted in social divisions of labour (Harry, Maltby and Szulecki 2024; Stevis 2020), I focus on the organizational level because this is where difference is productive of development and change. This is the level at which agency is enacted and ideological perspectives debated as labour organizations struggle to overcome divisions among workers, and between unionized workers, their communities and the body politik. I seek in this paper to bring out these dynamics, highlighting where, and under what conditions, labour actors have confronted and addressed barriers to more effective action.

Québec is an interesting case from which to theorize these dynamics. Electricity generation is already decarbonized and nearly fully unionized. We long ago met Huber's (2022) call, issued in a US context, for the unionization and nationalization of the electricity network as a lever to decarbonization. In Québec there is less in the way of ‘low hanging fruit’, as one close observer put it, and climate action has turned to electrifying transport and decarbonising industrial processes. Here, the focus turns to skills retraining, and bargaining strategies over investment and technological change, while questions arise around social priorities. The construction sector in Québec has been at full employment for over a decade and union membership in commercial and industrial building is compulsory. The ‘good green jobs’ strategy pushed by the building trades across North America (Figueroa 2017; Nugent 2017) is consequently of little strategic interest. There is no fossil fuel industry to fund climate change denial, which is found only on the margins of political debate, and has no expression within the labour movement. The environmental movement in Québec is amongst the strongest on the continent, well organized with deep roots in popular consciousness and a number of recent victories to its credit.

Putting this together, we are confronted with labour climate action beyond the ‘obstruction’ versus ‘green jobs’ dynamic which prevails across the rest of Canada and the United States (Vachon 2023). In Québec, the labour movement is concerned with the second-order problem of concretizing climate action at work, with mobilizing workers behind union climate positions in order to create a *rapport de force* with employers and the state, and with mediating between vying social visions of a zero-carbon economy. Higher levels of unionization, with ‘social dialogue’ in the high-profit and high-emitting sectors, combined with legacies of corporatism more broadly, provides organized labour with greater opportunity to address climate at the bargaining table and, to a lesser extent, within the state. Labour needs a ‘seat at the table’, but the Québec case forces us to confront the question, ‘in order to demand what?’ The Québec case should be read as a cautionary tale, consistent with the findings of Scandinavian research : stronger institutional and associational power does not resolve internal barriers to more effective labour climate action that may be less apparent in cases of labour and environmental weakness.

In what follows I address these questions by, first, briefly placing labour's response within the context of Québec's carbon emissions profile and government climate policy. The paper describes the evolution of the major union federations on climate change and profiles initiatives that have been taken over the past several years. I then turn to a discussion of the tensions and contradictions that have arisen within unions and between unions in different sectors engaged in climate action, and the ways in which these are being addressed. A final section concludes with a commentary on future challenges for labour climate action.

## Methodology

I have relied on two sources of primary information. Three years ago the School of Industrial Relations at Université de Montréal offered its first, ongoing graduate-level course on « La crise climatique et l’avenir du travail » (*The climate crisis and the future of work)*.^1^ When developing our class, my colleague Mélanie Laroche and I spoke extensively with unionists and allies who have been involved in labour climate action in Québec. Secondly, and more substantively, I have drawn on the formal presentations and discussions that animated our class in 2022 and 2023, which was based largely on invited participants speaking to their organizational and workplace experiences engaged in climate advocacy and activism. Panels were formed by industrial sector (mining, metal processing, construction, public sector), and questions were posed across sector, organized by theme (collective bargaining, member mobilization, political action). Panelists were asked to respond to questions relayed ahead of time, and developed their answers in classroom exchange.

The class heard from all labour climate actors that we were able to identify through contacts with key leaders across Québec's fractured labour movement. Participants are listed according to rank within union hierarchies and across economic sectors in Table 1. I have anonymized all contributions, making in-text reference solely to union rank, role, and economic sector. This version of the paper has been circulated among all invitees for their comment and consent.

**Table 1.** Role and Sectoral Distribution of Union Participants in Years 1 (2022) and 2 (2023), cummulative.

	Metal mining and processing	Manufacturing	Construction	Public Sector	All sectors
Federation president/vice-president	2	2		1	
National union president		2			
Local union president	2				
Federation staff			1	1	9
National union staff	3			2	
Union members				5	

This classroom-based methodology is limited with a view to bringing out the full range of climate perspectives within labour organizations. Importantly, our understanding of member perspectives was mediated by union officials. Absent were rank-and-file workers in high-emitting industries, whose views on climate were represented to us by union staff and leadership. We were able to hear from front-line workers in the education sector. However, these were climate champions whose views were, by their own admission, not held by a majority of their colleagues. We have no reason to doubt the veracity of perspectives imputed to members, especially as no dissensus emerged on the matter across union organizations and positions within union hierarchies. Further research is being conducted on member mobilization in high-emissions industry employing workplace visits and interview-based methods.

## Emissions Profile and Climate Policy in Québec

The Québec government began calculating emissions in 1990 when the first emissions reduction targets were set. Emissions have remained fairly constant, falling after the 2008 crisis from a 2003 peak of 89,6 mega tonnes CO2 equivalent to a pandemic low of 76,2 mega tonnes in 2020, after which emissions rebounded with the lifting of pandemic measures.^2^ Electricity generation accounts for a mere 0,3 percent of current emissions. The transportation sector remains overwhelmingly reliant on fossil fuels and represents the single largest source of emissions, at 43,3 percent. Industry accounts for 29,4 percent. Commercial, residential and institutional buildings account for 10 percent and agriculture for 9,2 percent. It bears noting that commercial trucking is counted under transportation rather than industry. If these emissions are added to the industry column, along with agriculture and industrial waste, the for-profit sector of the Québec economy would account for 62 percent of total emissions. Well over half of the province's emissions are produced in the workplace as a part of the labour process, not including the commute.

Government climate policy can be characterized as passively supportive of a technology-led transition and market mechanisms consistent with the pursuit of ‘green capitalism’ (Caron 2015). This policy orientation is held in consensus among the dominant political parties. Québec was an early adopter of market-oriented climate policy. Beginning in 2013, the province's main industrial and commercial emitters have been covered under the Western Climate Initiative's (WCI) cap-and-trade system. Apart from the many criticisms that can be made of cap-and-trade schemes and the underlying economics (Vlachou and Pantelias 2017), carbon prices set on this market are too low to drive the transition.^3^

Current government policy as outlined in the *Plan pour une économie verte 2030* (Plan for a Green Economy - PEV) is heavily focused on decarbonisation of road transportation, with a ban on the sale of ICE private cars by 2035, consumer rebates for EV purchases expiring in 2027 and public investment in charging stations. The plan promotes the exploitation of the province's vast mineral wealth by multinational capital, a repackaging as climate policy of long-planned public investments to spur mining development. Government financial support in the hundreds of millions of dollars for the construction of battery plants and industrial decarbonisation has taken the form of equity stakes, low-interest loans, and outright grants. The lack of transparency by which this support is granted, and in one case expedited regulatory approval of a now failed major project, has proven controversial, and has been denounced by primarily public sector unions on procedural and good governance grounds (Shields 2024). Government financial supports are granted on condition of meeting emissions targets but are accorded without employment conditionality, either in terms of jobs created, wages or union rights. Industrial unions take a ‘you build it and we will organize it’ approach. The position of the *Fédération des travailleurs et travailleuses du Québec* (The Québec Federation of Labour - FTQ), the main private sector labour central, is that any public moneys invested in decarbonisation measures in private enterprise must be accompanied by comparable firm investment, job guarantees, and the establishment of a labour-management committee overseeing plant-level transition plans (FTQ 2019, 24). However, the absence of just transition legislation – or any significant labour or welfare state reforms accompanying such ‘green’ industrial policies – limits the institutional power of unions to ensure that newly created ‘green’ jobs are decent jobs. Government policy assumes ‘good jobs’ will be created in the course of multinational corporate investment in ‘green’ industries.^4^ To land these investments, public agencies promote the province's decarbonised energy grid, access to key minerals, and low wages relative to North American standards (Gerbet 2024).

Industrial decarbonisation in Québec will have important consequences for a largely unionized workforce. The largest industrial emitters are found in a relatively small variety of sub-sectors, the largest of which include cement, oil refining, aluminum, steel and pulp and paper. Emissions in these workplaces derive from the burning of fossil fuels as a source of energy, as well as from industrial processes. The latter are ‘difficult to abate’ as zero-emissions production is technically feasible but considered not to be commercially viable, in other words, the necessary investments are not profitable or insufficiently profitable. The five sub-sectors listed above form an important part of the province's economic base, including nearly 20 percent of total exports.^5^ These high-emissions industries also tend to be strongly unionized and employment is relatively well paid. Of the twenty highest emitting worksites in Québec, representing 20 percent of total emissions, all but one is unionized. Eight are unionized with Unifor, another eight with the Métallos (Steelworkers), and two with the FIM-CSN (*Fédération de l’industrie manufacturière, Confédération des syndicats nationaux*).

## Québec Labour Confronts the Crisis

Labour climate action in Québec is driven by a relatively small group of highly motivated salaried staff, union leadership and rank-and-file activists. The staff is on the Left, university-educated and well-read in radical climate literature. They are typically located in the socio-political action departments of their respective unions and the climate crisis represents one file among others that they manage. The largest union centrals will have at most one full-time position dedicated to climate. The largest building trades federation in the province, with 90,000 members, has one staff rep working on climate, amongst other files. These staffers form an epistemic community and work together across union divides. They are somewhat isolated within their organisations and have little control over union policy. Commitment to climate action among union leadership is uneven, especially at the local level. There is greater awareness at the national or federation level of the main industrial unions. “When we look at the list of the highest emitting work sites in Québec, I count how many we represent and realize that we have a problem” (national union president, metal and mining). In the public sector, climate action is pushed from below by rank-and-file climate activists in the education sector, and by union staff at the national union level.

Labour climate leaders perceive themselves to be ‘playing catch-up’ with respect to Europe, the main reference point, but ahead of their colleagues in the rest of Canada. There has been little action on climate prior to 2014. Québec labour's engagement on environmental issues more broadly has a longer pedigree. The FTQ passed its first resolution on environmental sustainability in 1962 (Bernier 2020). Revisionist historiography of industrial unionism in Québec has unearthed an ecological current going back to the 1950s, including strike demands on health and safety issues related to working with toxic materials, as well as broader concerns over pollution in working class communities (Bécot 2015). CSN unions began lobbying government to reduce industrial runoff in the early 1960s, supported Canada signing the Kyoto protocol in 1999 and called for green industrial policies and investment in public transportation to meet emissions targets. The CSQ (*Centrale des syndicats du Québec*), representing mostly education workers, made a push in the 1990s to foreground ecology in the curriculum of the public school system (Bernier 2022). On climate, the FTQ, more exposed to climate action given its stronger representation in high-emitting industries, has taken the lead among union centrals, followed by the CSN and CSQ, much more rooted in the public sector, with the CSD further behind.

Union centrals were challenged to define their climate position in 2014 with the emergence of a powerful grassroots campaign against a proposed oil pipeline traversing southern Québec. Trans-Canada's Énergie Est would have expanded tar sands production in Alberta by increasing access to the European market via a port terminal on the lower St Lawrence. *Coule pas chez nous* (Don’t leak around here) turned public opinion against the project and ultimately defeated it. Although not affiliated to the coalition, most union centrals and some large public sector unions came out against the project. The CSN and SFPQ (*Syndicat de la fonction publique et parapublique du Québec –* Union of public and para-public civil service*)* were more forthright in their support of the coalition but less exposed to the employment dilemma (Pineault 2016). The FTQ's position is noteworthy given that Trans-Canada had won the approval of powerful FTQ affiliates, including the building trades and Unifor, which represent pipefitters and refinery workers (Shields 2016). Along with Unifor-Québec, the FTQ had given its approval to a previous pipeline proposal on the grounds that it would have supported refinery jobs in Montréal East (FTQ 2013). In its submission to the regulatory body considering the merits of the Énergie Est project, the FTQ now argued against the idea that there was an employment dilemma at issue. The pipeline would not create significant long term employment, would threaten more sustainable employment growth in ‘green’ areas of the economy, and would undermine the credibility of the province's climate pledges.

Out of this experience came a willingness among unions, environmental groups and community organizations to better coordinate their work, to bridge divides between unions and ENGOs, and to avoid rifts between unions. At meetings held between FTQ leadership and Greenpeace staff on the margins of the Ottawa Social Forum, the “FTQ agreed to talk more about climate justice and Greenpeace agreed to talk more about jobs and the economy” (Common front organizer). The *Front commun pour une transition énergétique* was formed in 2015, at first with 30 and now 80 affiliated groups claiming a combined membership base of 1,8 million. It includes all 4 union centrals and 5 large unaffiliated unions. *Front commun* is a notable choice of words. It refers both to a united negotiating position across Québec's labour movement as well as the associated 1972 public sector labour dispute, which took on the character of a general strike that defied back-to-work legislation and prison sentences given out to labour leaders (Rouillard 2004). It represents the highpoint of class struggle in Québec, analogous to the New Deal years in the United States insofar as it is invoked today as a touchstone for the climate justice movement. The use of the term by environmental organizations should be understood as a plea for unity while also conveying to union allies that climate activists understand the importance of workers power to effect the climate action that is required. The basis of unity refers to a common understanding of the imperative of moving rapidly towards a zero-carbon economy and systematic social change guided by social democratic, feminist and de-colonial politics (www.pourlatransitionenergetique.com). Eschewing the language of ‘just transition’ – too narrow in application and timid in scope – the common front refers to *une transition porteuse de justice sociale* (a transition that will bring social justice). In other words, the fight for a zero-carbon economy must result in a more equal, participative and inclusive society. The coalition has produced a 120-page road map detailing this vision, sector by sector. The *Front commun* has a significant legislative win to its credit, a 2022 law that bans fossil fuel exploration on Québec territory and mandates the shutdown of existing wells, one of the first jurisdictions in the world to do so.

In 2020, the FTQ joined a more selective, neo-corporatist coalition that was called on by government to provide post-pandemic policy advice. The G15 + brings labour, social economy actors and business elites in ‘green tech’ together through a ‘social dialogue’ and ‘social cohesion’ lens on the energy transition (Viens, Sapinski and Laurin-Lamothe 2024). The historical touchstone here is the social partnership model that emerged in Québec in the 1980s as the front commun years gave way to neoliberalism. Social dialogue remains extant within consultative, neo-corporatist institutions in Québec that are tasked with advising government on skills and regional economic development, and is grounded in cooperative labour relations in high-profit sectors such as mining and metal processing. G15 + communiqués and government submissions stress the importance of skills and training, strategic investments in public services including mass transit, child care and housing, the circular economy, and social dialogue, to ensure inclusive growth and to maintain economic competitiveness (https://g15plus.quebec/vision). Labour's lobbying for tripartite just transition legislation, with representation from ENGOs, labour and business, has been as yet unsuccessful beyond non-binding motions in the national assembly.

Finally, labour climate staff have organized a network exclusive to organized labour, the *Réseau intersyndical pour le climat* (Inter-union climate network - RIC), which includes all 4 centrals and 5 large unaffiliated public sector unions. The RIC was formed online during the pandemic and in response to the historic 2019 climate strike that brought 500,000 mostly young people to march through Montréal streets on a Friday afternoon (Baillargeon and Shields 2019). The climate strike was organized by a network of workplace climate activists, outflanking the unions. In the RIC, labour “wanted to have our own thing” (union federation staff) where union leadership and staff could discuss internal divisions in caucus prior to meeting with government. Rivalrous labour centrals have been able to ringfence the climate file and present a unified political position. The RIC also manages private-public sector disagreements which have not yet come into the open. Strategies and tips are shared on how best to engage members on the climate crisis, inter-union committees have been struck on different facets of labour climate action, including pension divestment from fossil fuels, and the network regularly produces webinars that are open to the public along with workshops for affiliated unions.

## Tensions and Contradictions

In the following I discuss 3 areas of tension and conflict that challenge labour climate action. These areas have been identified by labour climate actors and have been recurrent in seminar presentations and discussion. These are treated separately for ease of exposition. These areas are : 1) tension between union leaderships and activist layers, on the one hand, and union membership, on the other, with respect to the importance that climate action ought to occupy in union strategy; 2) sharp tensions within the labour movement on strategic and tactical questions having to do with rank-and-file organizing, industrial legality, and political demands; 3) an emerging contradiction between public sector positions on climate action and sustainable futures versus a focus in high-emitting industry on technological fixes and a narrower conception of just transition.

### Between Leadership/Activist Layers and the Membership

Union staff, national leaderships and rank-and-file organizers all agree that the most significant barrier to more effective union climate action lies in the difficulty of mobilizing members. “The first obstacle to [labour] climate action is our own members. They oppose jobs and climate action in their heads. And as long as that is the case, there will be no movement” (union federation staff). At the same time, all understand the importance of workers taking ownership of the issue. “What's important is that workers have a capacity to collectively come up with their own position. This is important for the workers involved but also for the transition itself, or it will fail” (federation president, manufacturing sector). Membership mobilization is a complex problem with many strands. In high-emissions sectors, staff argues that workplace change is inevitable regardless of scenario. An oft repeated slogan is “either you are at the table or you are on the menu”. An unfortunate corollary of this is that climate action is framed as a workplace threat against which workers will need to act pre-emptively. “We need to get ahead of the issue” (union federation president) does not mean pushing for stronger climate policy. The FTQ has found some purchase with this argument in the natural gas industry, where workers have agreed to set up a ‘climate lab’ to workshop decarbonization and transition measures, “because they know that otherwise they will lose their jobs” (federation staff). The climate lab initiative is intended to raise consciousness and create capacity at the workplace level to negotiate the transition, case studies that will then be used in lobbying (FTQ 2022). The CSN is pushing to have every local establish an environmental committee (www.vertlasolidarite.org).

In metal processing, workers have been more skeptical and resistant to talk of transition. “When they hear ‘just transition’, they think we are organizing them out of a job. They want us to organize to keep their jobs” (local union president, mining and metals processing). When workers themselves raise the question of climate change in informal discussions with their representatives, it tends to be younger workers who relate climate change mitigation policy to their employment security. Workers committees in this industry are being established “only where the employers want them. The employers are more interested in talking about climate change than our members” (national union president, mining). Both Unifor and the Métallos have succeeded in voting a motion at their 2022 congress to establish these committees. Where a willingness to engage in climate action exists there is confusion about what the demands should be, and how to make climate change a concrete issue for the purposes of collective bargaining. Unionized workers have strong contract language against technological employment loss, which is likely to be how decarbonisation will affect employment levels in high-emissions workplaces. Workplace health and safety activism around toxic materials is less helpful here as the ecological consequences of workplace emissions have a much less immediate effect on workers bodies and the health of their communities. Unions across sectors do recognize climate change as a health and safety issue and anticipate having to negotiate workplace heat protocols in the near term (local union president, metal processing).

In the public sector, climate activists have struggled all the more with the difficulty of making concrete and immediate a global phenomenon that is removed from the labour process. The climate crisis has not yet become subject to collective bargaining. Climate was not addressed during the common front of 2023, which saw 550,000 workers picketing over a period of several weeks. At the negotiating table, union negotiators did float several ‘green clauses’ : subsidized transit passes, a change in reimbursement policy to include public transportation, and the establishment of joint workplace committees to oversee decarbonisation. That the employer refused to discuss even such modest and cost-neutral demands was owing, in the estimation of one of the negotiators, to ideological reasons (federation president, public sector). The treasury had already pronounced in a previous round that pension fund investments in fossil fuels would not be subject to negotiation. But it is closer to the mark to recognize that climate gets pushed down the list of bargaining demands by members in assembly or when polled individually. Some such demands as the greening over of parking spaces at cégeps (colleges), or restrictions on air travel in the university sector, would involve the union bargaining away a perquisite. As CSQ staffer Dominique Bernier notes, forty years of neoliberalism, wage stagnation, increased precarity and indebtedness have pressured public sector unions to focus on wage demands (Bernier 2020). The climate crisis will only add to this pressure. The 2023 common front strikes can already be understood, at least in part, as an attempt to maintain wage levels against the inflationary effect of ecological breakdown (see Wallace 2020 on Covid-19; on the inflationary consequences of climate change, see Kotz, Levermann and Wenz 2024). As the climate crisis will increase public sector workloads and stress, the same can be said for pressures on union demands around working conditions. Public sector strikes are necessary to defend the public services that climate resilient communities will require.

Insofar as climate has been a political issue before becoming a workplace issue, members’ positions are subject to political allegiances along with modes of life, residential location and leisure pursuits that are not likely to be politicized in ways that align with climate job consciousness. Labour climate staff and activists work to bring discussions back to a workplace interest. One climate communicator in the building trades begins his presentation on climate with a picture of a Ford 150 pickup, tells his audience “I’m not here to take your truck away from you”, and then proceeds to discuss work opportunities in a green transition (federation staff, construction). A workplace activist in the cégep system frames climate as a question of professional ethics and in terms of pedagogical values of inter-generational justice. Teachers experience ecological grief in having to face, every day in the classroom, young people whose futures have been foreclosed (workplace activist, education). Climate activism gives positive expression to this distress. A nurse intervening in an assembly in support of her union's position against an oil pipeline brings the issue back to her workplace by linking fossil capitalists to the governing political elites behind austerity. Leisure pursuits may provide some openings. Workers in the mining and metal processing industry typically have cabins in the woods where they go fishing and hunting with their families. They are not as alienated from nature as one might suppose from an abstract schema of commodified labour power.

In the high-emitting sectors most exposed to climate policy, climate consciousness correlates with position in the union hierarchy. National leaderships are concerned with the future of their organisation, which is linked to the future of the industry and the political capacities of the union to negotiate industrial change. They are pushing local presidents to take up these concerns. Workers’ skepticism of union capacity to negotiate a just decarbonisation of work is well-founded. The green transition under eco-modernist lines will be a capitalist restructuring of work, and this tends towards employment loss and de-skilling. De-industrialization and free trade, still within living memory, were unjust transitions, accompanied by broken promises of skills retraining and new economy jobs. This time, unions have less capacity to negotiate change and are visibly not included in policy making. The president of a regional labour council explains the dilemma:

Positioning ourselves for the just transition does not mean cutting jobs but transforming jobs. And they [members] don't see it because there are no measures put in place by governments. Governments do not actually believe in the just transition. And when there are debates at that level, I will take for example the woodland caribou issue. The union organizations that are most impacted are Unifor and the Steelworkers. For years, the unions have been asking to be included in the discussions with indigenous people, employers, citizen groups to make their voices heard because it is they who work in the woods with them and who are confronted precisely with this problem. And they are excluded. So how can you bring the position of just transition to workers when workers see that you are not even invited to the table?

### Strategic and Tactical Questions

As in Scandinavia, our industrial relations institutions in Québec were not established with a view to effectively channeling workers socio-political action through institutional channels. With climate action largely blocked in collective bargaining, and labour's political lobbying falling on deaf ears, workplace climate activists have taken initiative on the margins of Québec's labour relations regime. *La planète s’invite au parlement* (the planet invites itself to parliament, LPSP), was formed by climate activists in the education sector in the context of a 2018 provincial election campaign that sidelined climate. With the support of the main labour centrals, student unions and environmental organizations, the group organized demonstrations in the tens of thousands in Montréal, Québec City and other municipalities demanding an end to fossil fuel exploration, all subsidies to the sector, and for a climate policy consistent with science and internationally agreed targets (Giguère 2018). In 2019 LPSP organized the historic September 27 climate strike in Montréal which, with the presence of Greta Thunberg, brought out more than a quarter of the city's total population. The 27 September strike represents the world's largest climate strike to date, the largest political demonstration in Québec history, and, in hindsight, the highpoint of the ‘Fridays for Future’ movement. LPSP activists struck their schools and colleges for the day, turning out most of the city's adolescent student population. Management decided not to contest what otherwise would very likely have been declared an illegal strike and agreed to close the schools.

The broad coalition headed by LPSP broke down over the strategic question of climate action and industrial legality. As noted above, the enormous success of September 27 and the promise of rank-and-file organizing pushed the labour centrals to form the RIC. “They freaked out”, according to one non-union observer. LPSP activists fell back to form an inter-union network based in the education sector, *Travailleurs.euses pour la justice climatique* (Workers for climate justice – TJC), which acts as a ginger group within education unions and their affiliated centrals, the CSN and the CSQ, to push for stronger climate positions. The group is guided by eco-socialist thought, linking the climate crisis to capitalism and centering working class agency. “We believe that unions are the only organized force capable of taking on the fossil fuel lobby” (https://justiceclimatique.org). Their practice is informed by the organizing approach championed by Jane McAlevey (2016), Labor Notes and by the experience of the Chicago Teachers Union.

The leadership of Québec's labour centrals and national unions strongly opposed moving forward with a social strike strategy, which is regarded as irresponsible and unrealistic in the near term. There was insufficient support for a political strike within most unions beyond higher education, and in the absence of broad-based support, union leaderships feared imposition of harsh financial penalties on union organizations and individuals. Union leaders in the industrial economy are quick to point to differences in the ways that workers are disciplined and working time is valued. TJC has since evolved to now include local unions as members (24) with a dues base that sustains 3 full time paid positions. A decision was made in Fall 2022 to not continue with climate strikes in the context of a hardening of employer and government threats and an understanding of the need to build more broadly, beyond education, to health services and the transportation sector, where worker assemblies are organized in order to “create dialogue between workers and researchers, discuss sustainable futures, and reflect on how to concretise labour strategy” (worker activist, public sector).

### Public Sector Versus High-Emitting Sectors

Climate action in public and private sectors are “two different realities” (labour federation staff). In the public sector, demands for stronger climate policy are pushed from below, aligned with activists at local and regional levels and the orientation of staff in political action departments. In the private sector, climate action is framed in reaction to climate policy and even to employer initiatives, and is driven from the top. In high-emitting sectors, multinational corporations welcome the establishment of joint climate committees in the expectation that they will smooth the anticipated restructuring of work. In the public sector joint committees are dismissed “with the back of the hand” (union federation staff).

Québec's public sector will buckle under the crisis. The municipal sector will be tasked with public health and safety, emergency relief and continuous infrastructure repair. Public sector buildings are old, were not built to withstand flooding, are poorly ventilated and unequipped to deal with extreme heat (union federation staff). Extreme heat and zoonotic disease spread by novel vectors will increase occupational illness (Adam-Poupart et al. 2021), and illness in general, taxing the health care system. Early childhood educators will be minding children inside cramped indoor spaces more often in the summer, already a reality in Montréal in 2024. An unknowable number of climate refugees will require housing and assistance accessing public services and employment. The experience of front-line public sector workers during Covid-19 opens a small window into the work stress that the climate crisis will bring in its train. Difficulty with public sector workforce retention due to precarious employment, overwork and low pay – the fruit of decades of austerity, only worsened by the pandemic – represents a major, and largely unacknowledged, climate liability.

Public sector labour staff anticipate these effects and present a strengthened public sector as a pillar of Québec's just transition. The transitioning of industrial workers calls for a vast expansion of public education and skills development in a context of widespread illiteracy. Job loss in high-emitting sectors will have to be compensated by growth of low-carbon public sector employment, targeted in single industry regions and towns disproportionately affected by climate policy. The SFPQ, which represents provincial civil servants, has called for increased state capacity to support cooperative agriculture, social housing and tenants’ rights, and inter-regional public transportation, all framed in terms of climate change mitigation and resilience. An often-invoked slogan in public sector labour climate discussions is “to make the climate transition a tool of social justice, and social justice the motor of the ecological transition”. This framing is aligned with eco-socialist or even de-growth perspectives, although labour staff is more likely to invoke ‘buen vivir’, a borrowing from Latin American social movements. “We need to promote socially useful and environmentally sustainable work, as opposed to work that responds to a need to make profits” (national union staff, public sector).

This future is flatly rejected by union leaders in industrial sectors : “we’re not going to be growing tomatoes” (local union president). “What we need are good-paying jobs in the regions”, where job transition with reskilling is a last resort. The largely private sector FTQ recognizes that the just transition cannot just be about “taking those white middle aged guys out of one industry to another industry, we need a gender balance” (union federation staff). But in the membership guide the central has produced on the just transition the importance of public services is not mentioned, and this author counts one passing reference to gender analysis. Climate change is explained as the result simply of emitting greenhouse gasses. There is mention of the need “to rethink our economic model at every level” (FTQ 2022, 14). The solution, a just transition, boils down to electrification plus workers committees.

In the metal processing industry, leadership across union divides wants to have members commit to climate strikes demanding that headquarters in London or Luxembourg invest in zero-carbon processes. They see this as necessary to secure employment and social acceptability, assuming that higher carbon prices and more stringent regulation are in the pipeline. This must be accompanied by carbon border adjustments to protect a national or continental ‘green’ market, without which “there will be no just transition in Canada” (local union president, metal processing).

Québec's forestry industry, itself a major emitter, is in crisis after 18,5 million hectares burned in 2022 and thousands of workers were laid off. Unsustainable practices over decades have transformed forests into fire-prone tree plantations and have pushed the woodland caribou to the brink of extinction (Bégin and Schepper 2020). CSN union leadership sees caribou loss as a “canary in the coal mine” moment (union federation staff), further sign that current practices will drive the industry to the wall: “We need to move now or we will lose the jobs. Once they cut the last tree in northern Québec, the companies will shut down and move to the US where there are private forests” (union federation president, manufacturing). The CSN manufacturing federation, the FIM-CSN, has allied with a major conservation organization, Nature Québec, along with indigenous nations, to push for a reduction in cuts and an overhaul of the province's forestry regime. Unions in the sector call for industrial policies to promote secondary and tertiary transformation of lumber as a means of dealing with climate-related job loss, along with retraining and employment insurance measures for a just transition (FIM-CSN 2023; Métallos 2024).

In outline we can see emerge two social blocs bi-secting the labour movement. An eco-modernist bloc including industrial unions and industrial capital in renewed tripartite arrangement with the nation state, and an eco-socialist bloc involving public sector labour and grassroots environmental organizations and citizens movements campaigning for climate justice. The former has more power, and we have seen it prevail in the United States as public investments in the caring economy were jettisoned in favour of the building trades and ‘green’ industries (Tufts 2025). The same can be said of Canada's flawed just transition legislation targeting mostly white and male workers in the coal sector (Mertins-Kirkwood and Deshpande 2019). Workers in industry have more power than public sector workers because they produce capitalist profit and because their demands for public supports are aligned with the interests of employers who are well-placed within the state. Public sector workers have less structural power at work, and less power within the political realm, because their employers – neoliberal governments – are committed to austerity and refuse to negotiate green investment. Public sector workers are demanding that climate policy align with climate science, while in the private sector, climate policy is framed as a threat against which unions must respond.

In Québec, the CSN is under the most pressure as the federation includes education workers, at the forefront of climate action, and industrial workers in high-emitting sectors, the most exposed to climate policy. These tensions have come out in CSN annual congress, with public sector and private sector union actors intervening on opposing sides of climate motions, and with one union federation threatening that if the CSN moves ahead with more activist positions, they will leave (union federation president). If unions in high-emitting sectors are able to bargain industrial decarbonisation with their employers, keeping to the polluter pays principle, then these tensions will abate. If, as seems more likely, industrial decarbonisation is paid for out of the tax base, through tax credits or grants, inter-union tensions will increase.

## Conclusion

To date, Québec's labour movement has shown an uncommon ability to overcome organizational conflict and differing union perspectives on climate action. It has been more open to climate coalition work with environmental organizations, including not only the large corporate ENGOs, but more activist grassroots groups as well. In several instances, we have seen how tensions within the labour movement are productive of evolution and positive change. In public statements, press communiqués and submissions to government review boards, tensions between the strategic vision of leadership, member fear of job loss, activist frustration over the slow pace of change, and between public and private sector unions, are resolved in the abstract through calls for more interventionist public policy, ‘green growth’ resolving employment dilemmas, and an inclusive and equitable just transition. Tensions are also offset and deferred to some extent by the practice of union federations joining coalitions linked to very different social projects, the full implications of which are not evident because none of these coalitions have built the power necessary to push the state to act decisively in one direction or the other.

What conditions underlie the ability of Québec labour organizations to cooperate above these sources of tension and conflict? Two moments stand out. In the first moment, a broad, grassroots ecological campaign to block the Énergie Est pipeline pushed union centrals to align with climate justice, in one case over the opposition of powerful affiliates. Large public sector unions began to engage more seriously with the politics of the climate crisis and took stronger public positions at this time. In the second moment, this same coalition now including rank-and-file worker activists organized the world's largest student strike for climate. This galvanized labour leadership to create the RIC and to invest more resources in climate action and member mobilisation. There is a widespread recognition, throughout the leadership and activist layer in the Québec labour movement, that coalition work will be necessary if labour is to exercise any political influence in the climate crisis. This is due to the weakness of labour, a relatively low capacity to mobilise members for political action, and the relatively high degree of ecological consciousness in Québec society. The ability of labour to negotiate workplace change in an era of ecological breakdown depends on maintaining close ties to environmental organizations and ecological activists. The threat of isolation and delegitimization keeps organized labour disciplined on climate.

But the climate crisis is on its own timeline. As Andreas Malm has written, “it really is nature that comes roaring back into society in a warming world, and it is time that flaps its wings as it does so” (2018, p. 77). Behind the backs of the social actors, the “progress of this storm” pushes organized labour's contradictions forward. If we had more time, the tensions and conflicts addressed in this article would matter less. They could be resolved to some extent within the terms of a more liberal, pro-growth policy framework, although this would still bracket the question of broader ecological limits to growth as well as questions of colonialism and global justice. But because we are twenty, thirty or forty years too late, it is more likely that we will have to deal in short order with significant organizational stress in the labour movement. How this will play out in the near future is a less important question than how to overcome these tensions. The way forward in Québec as elsewhere is for union organisations to invest more resources in member engagement on climate, and through this engagement identify alignments between worker grievances on the job, opportunities for building union power, and science-based climate policy. These alignments may be few, and variable, but they can neither be identified nor provide a impetus to mobilization except through a deeper engagement of the membership.

## Notes

1. The UdeM seminar, co-taught with my colleague Mélanie Laroche and with the assistance of Jonathan Michaud, was given in the context of a climate summer school organized by John Peters at UofT, with simultaneous sessions via video link and interpretation, financed by a SSHRC Connection Grant. In subsequent years the UdeM and UofT seminars were taught separately. The climate summer school also relied on CRIMT resources and the support in particular of Gregor Murray, Nicolas Roby, Francine Jacques, and Sandra Cordero.

2. Figures from the Ministère de l’environnement et la lutte contre le changement climatique (MELCC), 2024. Accessed on September 30, 2024 at https://www.environnement.gouv.qc.ca/changements/ges/index.htm

3. The Québec Finance Ministry estimated at the market’s inception that prices would have to be set between $79 and $93 per tonne ($93 and $115 in 2023 dollars) in order to induce the necessary firm investments (Purdon and Sinclair-Desgagné 2015, p. 11).

4. I owe the above points to Christian Pépin. A private member’s bill to fund a just transition was tabled in 2021 but not passed into law.

5. Author calculations based Stats Can data, accessed at https://statistique.quebec.ca/fr/produit/tableau/valeur-des-exportations-internationales-par-produits-quebec-et-canada
